# The Citrus Flavonoid Naringin Confers Neuroprotection Against Cerebral Ischemia by Enhancing Endogenous Antioxidant Capacity and Suppressing Neuroinflammation

**DOI:** 10.3390/foods15193449

**Published:** 2026-09-27

**Authors:** Ratchaniporn Kongsui, Nut Palachai, Araya Supawat, Sitthisak Thongrong, Jinatta Jittiwat

**Affiliations:** 1 Division of Physiology, School of Medical Sciences, University of Phayao, Phayao 56000, Thailand; ratchaniporn.ko@up.ac.th; 2 Faculty of Medicine, Mahasarakham University, Maha Sarakham 44000, Thailand; nut.p@msu.ac.th (N.P.); araya.su@msu.ac.th (A.S.); 3 Division of Anatomy, School of Medical Sciences, University of Phayao, Phayao 56000, Thailand; sitthisak.th@up.ac.th

**Keywords:** middle cerebral artery occlusion, ischemic stroke, oxidative stress, neuroinflammation, dietary flavonoid

## Abstract

Naringin is a naturally occurring flavanone glycoside found in citrus fruits that has been widely studied for its antioxidant and anti-inflammatory activities. The precise mechanisms by which naringin exerts protective effects against ischemic stroke are not yet fully understood. To address this gap, the current study examined the efficacy of naringin administration (100 mg/kg BW) in an experimental rat model involving permanent right middle cerebral artery occlusion (Rt.MCAO). Administration of naringin significantly improved neurological performance while reducing cerebral infarct volume and brain edema following ischemic injury. Histopathological evaluation further demonstrated that naringin preserved neuronal density in the cerebral cortex and hippocampus, indicating protection against ischemia-induced neuronal degeneration. In parallel, naringin attenuated glial activation, as evidenced by reduced expression of ionized calcium-binding adapter molecule 1 (Iba1) and glial fibrillary acidic protein (GFAP), accompanied by suppression of the pro-inflammatory mediators interleukin-6 (IL-6) and cyclooxygenase-2 (COX-2). Furthermore, naringin restored redox homeostasis by lowering malondialdehyde (MDA) levels and preserving the activities of the endogenous antioxidant enzymes catalase (CAT), glutathione peroxidase (GSH-Px), superoxide dismutase (SOD), and mitochondrial superoxide dismutase (mitochondrial SOD). A significant reduction in myeloperoxidase (MPO) activity further supported its ability to alleviate oxidative stress and inflammatory responses. Our findings indicate that naringin has neuroprotective effects in experimental ischemic stroke. These effects were associated with improved antioxidant defenses and reduced neuroinflammatory responses. However, further preclinical studies are needed to confirm the underlying mechanisms, optimal treatment conditions, and long-term effects.

## 1. Introduction

Stroke remains a leading cause of death and long-term disability worldwide, placing an immense burden on global public health systems [1,2]. This epidemiologic crisis is accelerating rapidly—particularly in developing nations—driven by an aging population and a steady rise in metabolic risk factors like high blood pressure, diabetes, obesity, and abnormal cholesterol levels [3]. For individuals who survive a stroke, the road ahead is often marked by chronic motor impairments, cognitive decline, and emotional instability, which severely diminish their personal independence and overall quality of life [4,5]. Beyond the immediate clinical impact, the socioeconomic toll is staggering, involving massive healthcare expenditures alongside indirect financial losses from lost workplace productivity and long-term caregiver stress [6,7]. Finding practical preventative measures and new therapeutic approaches is therefore a critical priority.

At the molecular level, an ischemic stroke triggers a destructive, self-reinforcing loop of oxidative stress and inflammation that drives progressive brain damage [8,9]. When blood flow to the brain is suddenly blocked, neurons are starved of vital oxygen and glucose. This energy failure disrupts the mitochondria (the cell’s power plants), triggering a massive, uncontrolled release of unstable molecules known as reactive oxygen species (ROS) [10,11,12]. The brain is uniquely sensitive to this kind of oxidative damage because it contains high amounts of fats and possesses relatively low natural defenses. Under normal conditions, a dedicated team of frontline antioxidant enzymes—specifically superoxide dismutase (SOD), catalase (CAT), and glutathione peroxidase (GSH-Px)—neutralizes these dangerous molecules. However, during a stroke, the flood of ROS quickly exhausts these enzymes, leading to widespread damage to cell membranes, vital proteins, and DNA, which ultimately kills the neurons [10,13]. This enzymatic failure and subsequent tissue damage quickly trigger the immune system, worsening the injury. This process activates a master inflammatory switch called nuclear factor kappa B (NF-κB), which commands the cell to produce harmful inflammatory chemicals like interleukin-6 (IL-6) and cyclooxygenase-2 (COX-2) that expand the damaged brain area [14,15,16]. At the same time, the brain’s resident immune cells, the microglia, transform into a highly aggressive state—a change marked by an uptick in a protein called ionized calcium-binding adapter molecule 1 (Iba1)—which amplifies the local tissue destruction [17,18]. Support cells called astrocytes also react defensively, swelling and increasing their expression of glial fibrillary acidic protein (GFAP) to form a physical scar around the injury zone [17,19,20]. Because oxidative stress and inflammation reinforce one another so tightly, effective therapies cannot simply target one isolated molecule; they must address this entire interconnected network.

From a nutritional standpoint, researchers are increasingly looking at natural, food-derived compounds to help reinforce the brain’s failing defenses before or immediately after an injury. One highly promising candidate is naringin, a natural antioxidant flavonoid found in abundance in citrus fruits like grapefruits and oranges [21,22,23,24,25]. A wealth of animal studies shows that naringin offers powerful protection to the brain during a stroke [26,27]. Recent studies, including reports published in 2024 and 2025, have continued to broaden our understanding of the biological effects of naringin in ischemic conditions. Experimental evidence indicates that naringin may limit ischemia–reperfusion-induced tissue injury, decrease infarct development, and support vascular function through the regulation of signaling pathways associated with oxidative stress, inflammation, and cell survival, including PI3K/Akt, NF-κB, and Nrf2 [28]. Beyond its cardiovascular effects, accumulating evidence suggests that naringin may also exert neuroprotective effects in cerebral ischemia, with potential benefits related to neuronal preservation, neurogenesis, and brain-derived neurotrophic factor (BDNF)-associated signaling [29]. These protective effects have been linked to attenuation of oxidative and inflammatory responses, preservation of endothelial and mitochondrial function, and reduction in cellular injury [30]. Nevertheless, most previous studies have focused on ischemia–reperfusion models, whereas the effects of naringin in permanent cerebral ischemia, in which reperfusion does not occur, remain less well defined. In particular, its effects on neurological function, brain edema, ischemic lesion development, neuroinflammatory responses, and endogenous antioxidant defenses following permanent focal cerebral ischemia require further investigation. Therefore, the present study examined the neuroprotective effects of naringin at 100 mg/kg body weight in rats subjected to permanent right middle cerebral artery occlusion (Rt.MCAO). Neurological function, brain water content, and ischemic lesion size were evaluated together with changes in neuroinflammatory markers and endogenous antioxidant defenses, including catalase (CAT), glutathione peroxidase (GSH-Px), superoxide dismutase (SOD), and mitochondrial SOD.

## 2. Materials and Methods

### 2.1. Experimental Compounds

In this study, dimethyl sulfoxide (DMSO) was used as the vehicle and was obtained from Thermo Fisher Scientific, Inc., Waltham, MA, USA (product code: D/4121/PB15). Piracetam, which served as the positive control, was purchased from GlaxoSmithKline (Bangkok, Thailand) Ltd. Naringin, a bitter flavanone-7-O-glycoside (C_27_H_32_O_14_; PubChem ID: 442428), was supplied by Chengdu Biopurify Phytochemicals Ltd. (Chengdu, Sichuan, China). The purity of naringin was verified to be ≥98% as determined by high-performance liquid chromatography (HPLC).

### 2.2. Animals and Experimental Design

Healthy male Wistar rats (8 weeks old; body weight 250–300 g) were obtained from the Northeastern Laboratory Animal Center, Khon Kaen University, Thailand. The animals were housed in standard metal cages with five rats per cage under controlled environmental conditions, including a 12 h light/dark cycle, temperature maintained at 23 ± 2 °C, and relative humidity of 30–60%. Food (commercial pellet diet) and water were provided ad libitum. All experimental procedures involving animals were performed in accordance with the guidelines of the Institutional Animal Care and Use Committee of Khon Kaen University, Thailand, and were approved under protocol number IACUC-KKU-1/68.

A total of 60 rats were randomly allocated into four experimental groups (n = 15 per group). Group I (Sham operation): Rats underwent a sham surgical procedure without further treatment. Group II (Rt.MCAO + vehicle): Following induction of focal cerebral ischemia by right middle cerebral artery occlusion (Rt.MCAO), rats received an intraperitoneal (i.p.) injection of 1% dimethyl sulfoxide (DMSO), which served as the vehicle for dissolving the test compound, for seven consecutive days. Group III (Rt.MCAO + piracetam): Rats subjected to Rt.MCAO were administered piracetam intraperitoneally at a dose of 250 milligram/kilogram body weight (mg/kg BW) for seven days. Piracetam was used as a positive control, and the selected dose was based on our previous study [31]. Group IV (Rt.MCAO + naringin): Rats received intraperitoneal injections of naringin at a dose of 100 mg/kg BW for seven days following Rt.MCAO induction. The naringin dose was selected based on findings from our in-house preliminary experiment, together with supporting evidence from previous studies [32,33]. The first doses of vehicle, piracetam, and naringin were administered immediately after completion of MCAO induction, and treatment was continued once daily for 7 consecutive days. The i.p. route was selected instead of oral gavage primarily for animal welfare considerations. Because the surgical procedure involved a cervical incision, oral gavage could require additional manipulation and extension of the neck during the postoperative period. Such handling may increase discomfort, place mechanical stress on the surgical wound, and cause unnecessary stress during recovery. Therefore, the i.p. route was considered a more appropriate method of drug administration for these postoperative animals. The 7-day treatment period was chosen to examine the effects of naringin during the early post-ischemic phase, when oxidative stress and neuroinflammation contribute to secondary neuronal injury. Cerebral ischemia promotes reactive oxygen species generation and glial activation, which may overwhelm endogenous antioxidant defenses and exacerbate tissue damage. Thus, the 7-day regimen was used to determine whether naringin could attenuate oxidative and inflammatory responses and thereby protect against neuronal injury following permanent Rt.MCAO. Moreover, within each experimental group, the 15 rats were further divided into three independent subsets of five rats (n = 5 per subset) according to the planned terminal assessments. The first subset (n = 5 per group) was used for molecular and biochemical analyses. Biochemical assays were performed to quantify MPO activity, MDA content, and the activities of the antioxidant enzymes CAT, GSH-Px, SOD, and mitochondrial SOD in the cerebral cortex and hippocampus. In addition, the protein expression of IL-6 and COX-2 was evaluated by Western blot analysis. The second subset (n = 5 per group) was used for immunohistochemical evaluation of neuronal density, astrocytes, and microglia in the cerebral cortex and hippocampus. The third subset (n = 5 per group) was used for neurological assessment, TTC staining, and brain water content. Neurological function was assessed longitudinally in these animals, followed by TTC staining to determine cerebral infarct volume. Brain water content was also measured using the same animals in this subset. Thus, each major experimental endpoint was evaluated using five independent animals per group (n = 5), representing biological replicates.

### 2.3. Middle Cerebral Artery Occlusion-Induced Focal Cerebral Ischemia Model

Rats were deprived of food overnight before surgery while continuing to receive water ad libitum. Anesthesia was initiated with 5% isoflurane and maintained with 1–3% isoflurane in 100% oxygen. The animals were continuously observed during surgery for general anesthetic and physiological status. Permanent focal ischemia in the right middle cerebral artery (Rt.MCAO) was induced using an intraluminal filament approach. A silicone-coated 4-0 filament was introduced through the internal carotid artery and advanced approximately 17 mm until gentle resistance was felt, which was used as the predefined anatomical criterion for MCA occlusion [34]. As an additional anatomical check, at the end of the experimental period, the brains were removed during perfusion and photographed to record the location of the occluding filament. The photographs showed that the filament was consistently located at the expected site of the middle cerebral artery, supporting successful and consistent MCA occlusion among the experimental animals. In the sham-operated group, the same vessels were surgically exposed, but the occluding filament was not inserted. Postoperative care began with immediate wound closure and topical antisepsis using 10% povidone–iodine. Post-surgical health was tracked systematically, with routine checks for dehiscence, bleeding, dehydration, dyspnea, abnormal motor or behavioral responses, and signs of prolonged distress under the guidance of veterinary staff. Criteria for humane euthanasia included acute body weight loss exceeding 20%, profound lethargy, wound infection, clinical dehydration, labored respiration, hemorrhage from natural orifices, sensory unresponsiveness, or unremitting distress. No subjects met these intervention thresholds, and all survived until their designated experimental endpoints.

### 2.4. Neurological Outcome Evaluation

Neurological function was evaluated at 24 h and 7 days after Rt.MCAO induction using a 20-point modified neurologic severity score (mNSS), with scores ranging from 0 to 20. The method was based on our previous publication [35]. Increasing scores corresponded to greater neurological impairment. A detailed description of the scoring criteria and grading scale is presented in Table 1.

### 2.5. Evaluation of Brain Water Content

Brain water content was evaluated using the conventional wet–dry weight method [35]. Following sacrifice, the whole brains were carefully removed and immediately weighed to determine the wet weight. The brain tissue was subsequently placed in a drying oven (Memmert GmbH, Schwabach, Germany) at 70 °C for 24 h and weighed again to obtain the dry weight. Brain water content was calculated as follows:

Brain water content = ((wet weight − dry weight)/wet weight) × 100

### 2.6. Measurement of Cerebral Infarct Volume

Cerebral infarct volume was determined using the 2,3,5-triphenyltetrazolium chloride (TTC) staining procedure described in our previous study [36]. Briefly, the animals were transcardially perfused with ice-cold phosphate-buffered saline (PBS), after which the brains were carefully removed. The isolated brains were coronally sectioned into 2-mm-thick slices using a standardized brain matrix. The sections were subsequently incubated in 2% TTC solution prepared in PBS at 37 °C for 30 min to distinguish viable tissue from infarcted regions. After staining, digital images of the brain sections were captured, and the infarcted areas were quantified using ImageJ^®^ software (version 1.53e, National Institutes of Health, Bethesda, MD, USA). The infarct volume (%) was calculated as follows:

The infarct volume (%) = [(contralateral hemisphere volume − non-infarcted ipsilateral hemisphere volume)/contralateral hemisphere volume] × 100

### 2.7. Assessment of Neuronal Density by Cresyl Violet Staining

Brain tissues were sectioned coronally into serial slices with a thickness of 30 μm, encompassing the cerebral cortex and hippocampus. The sections were stained with cresyl violet acetate (MilliporeSigma, Burlington, MA, USA) at 60 °C for 16 min to visualize Nissl-positive neurons for subsequent quantification. Histological examination was performed using an Olympus CX23 light microscope (Olympus Corporation, Tokyo, Japan) at 40× magnification. Representative images were captured from predefined stereotaxic coordinates according to a previously published protocol [37]. Neuronal density was assessed by identifying and counting viable neurons with preserved cellular morphology. For each cortical region of interest, three consecutive microscopic fields without overlap were examined in every section. The measurements obtained from these fields were averaged for each animal, and neuronal counts were expressed per 255 µm^2^, corresponding to the standardized area defined by the calibrated microscopic grid. To facilitate comparison between experimental groups, the resulting values were normalized against those of the sham-operated controls and expressed as a percentage of the sham group (% of sham).

### 2.8. Immunohistochemical Analysis of Microglia and Astrocytes

Immunohistochemical staining was conducted using 30-µm-thick, free-floating, paraformaldehyde (PFA)-fixed sections according to the methods established by Thongrong et al. (2023) [38]. To eliminate endogenous peroxidase activity, the tissue sections were initially rinsed and then incubated in 3% H_2_O_2_ followed by a blocking step with 3% horse serum (16050130, Gibco, Thermo Fisher Scientific). The sections were then treated with primary antibodies, mouse anti-Iba1 (1:250, MABN92, Sigma-Aldrich; Merck KGaA, Darmstadt, Germany) and anti-GFAP (1:300, MAB5628, Sigma-Aldrich; Merck KGaA). Following a series of washes in 0.1 M PBS, a biotinylated donkey anti-mouse secondary antibody (1:500, 715-065-150, Jackson ImmunoResearch Europe, Ltd., Strate, UK) was applied for 2 h at room temperature. Signal amplification was achieved through incubation with extravidin peroxidase (1:1000, E2886, Sigma-Aldrich; Merck KGaA). Visualizing the immunoreactivity involved a 10-min nickel-enhanced DAB reaction. The process concluded with mounting the sections onto positively charged slides, followed by dehydration in ascending ethanol concentrations, xylene clearing, and final coverslipping. Quantification of microglial and astrocytic expression was performed on DAB-stained sections using ImageJ (v1.53, NIH). Micrographs were acquired through a Nikon Ni-U microscope equipped with a 10× objective and NIS Element software (v5, Nikon, Tokyo, Japan). The selected regions of interest were specifically the cortex and hippocampal CA3. An automated thresholding technique was applied to convert positive immunoreactivity into black pixels. The software then calculated the area fraction of these pixels, and the results were reported as the percentage of total immunoreactivity for statistical comparison.

### 2.9. Protein Measurement

The total protein content within the cortical and hippocampal tissues was quantified utilizing the Lowry assay, strictly adhering to previously established protocols [39]. To ensure accurate calibration of the results, a standard curve was generated using bovine serum albumin (MilliporeSigma).

### 2.10. Determination of Myeloperoxidase (MPO) Activity

The activity of MPO in the cortical and hippocampal tissues was evaluated using a commercial colorimetric assay (MilliporeSigma, MAK068), as described in our previous report [35]. Briefly, the soluble protein fraction was obtained by tissue homogenization followed by high-speed centrifugation. Equal volumes of the sample and reaction mixture were incubated at room temperature in the dark. The reaction was terminated at 30, 60, and 120 min, followed by a final incubation with 5-thio-2-nitrobenzoic acid (TNB) reagent. Absorbance at 412 nm was measured and compared with a standard curve generated using serial concentrations of TNB. The final values were normalized to the protein concentration of each sample and expressed as units per milligram of protein (unit/mg protein).

### 2.11. Measurement of Malondialdehyde (MDA) Level

Malondialdehyde (MDA), a well-established biomarker of lipid peroxidation, was quantified to evaluate oxidative stress in brain tissue samples. MDA levels were determined using the thiobarbituric acid (TBA) assay (MilliporeSigma) following the protocol described by Ohkawa et al. [40]. The concentration of MDA was normalized to the protein content of each sample and is presented as nanomoles per milligram of protein (nmol/mg protein).

### 2.12. Measurement of Catalase (CAT) Activity

Following the procedure outlined by Goldblith and Proctor [41], the activity of CAT was quantified. Briefly, to prevent protein degradation and maintain enzymatic integrity, brain samples were homogenized in ice-cold phosphate buffer. Centrifugation of the homogenates was subsequently carried out at 10,000× *g* for 10 min at a temperature of 4 °C, after which the resulting supernatants were collected for CAT activity analysis. The reaction mixture consisted of the supernatant, phosphate buffer, and hydrogen peroxide (H_2_O_2_) as the substrate. The decomposition of H_2_O_2_ was monitored by measuring the decrease in absorbance at 515 nm using a spectrophotometer. CAT activity was normalized to the protein concentration of each sample and reported as units/mg protein.

### 2.13. Measurement of Glutathione Peroxidase (GSH-Px) Activity

Glutathione peroxidase (GSH-Px) activity was determined using a commercially available assay kit (MilliporeSigma, cat. no. MAK437-1KT) according to the manufacturer’s instructions. Briefly, brain tissues were homogenized, and the homogenates were centrifuged at 10,000× *g* for 10 min at 4 °C. The resulting supernatants were collected and used for enzymatic analysis. The assay mixture contained the tissue supernatant, phosphate buffer, glutathione reductase, nicotinamide adenine dinucleotide phosphate (NADPH), and hydrogen peroxide (H_2_O_2_). GSH-Px activity was determined by monitoring the oxidation of NADPH, reflected by the decrease in absorbance at 340 nm using a spectrophotometer. Enzyme activity was calculated from the rate of absorbance change over time and normalized to the protein concentration of each sample. The results are represented as units/mg protein.

### 2.14. Measurement of Superoxide Dismutase (SOD) Activity

Cortical and hippocampal SOD activity was determined using a commercially available assay kit (MilliporeSigma, cat. no. 19160-1KT-F) following the manufacturer’s instructions. All assay reagents were freshly prepared immediately before use. For the analysis, reactions were assembled in a 96-well microplate. The reaction mixture consisted of substrate solution and reaction buffer, after which mitochondrial samples, standards, and control preparations were added to their designated wells. To initiate the enzymatic reaction, the detection reagent was added to each well, and the plate was gently agitated to ensure adequate mixing. The reaction mixture was then incubated at 37 °C for 20 min to permit color development. Absorbance was then measured at 450 nm using a microplate reader. Enzyme activity was normalized to the protein concentration of each sample and calculated as units/mg protein.

### 2.15. Isolation of Brain Mitochondria for SOD Activity in Mitochondrial Fractions Analysis

Following perfusion, the cerebral cortex and hippocampus were promptly dissected and processed for mitochondrial isolation according to a protocol previously established in our laboratory [34]. Briefly, freshly collected tissues were homogenized in mitochondrial isolation buffer and centrifuged at 1000× *g* for 2 min at 4 °C. The resulting supernatant was collected, while the pellet was re-suspended in 0.2 mL of the same buffer and centrifuged again under identical conditions. The supernatants from both centrifugation steps were combined and mixed with 0.07 mL of 80% Percoll. Subsequently, 0.7 mL of 10% Percoll was gently layered over the mixture to establish a discontinuous gradient. The preparation was centrifuged at 18,500× *g* for 10 min to isolate the mitochondrial fraction. The mitochondrial pellet was then washed by re-suspension in 0.7 mL of washing buffer followed by centrifugation at 10,000× *g* for 5 min. The purified mitochondrial fraction was finally re-suspended in washing buffer and stored at −80 °C until subsequent analysis.

### 2.16. Quantification of SOD Activity in Mitochondrial Fractions

SOD activity in mitochondrial fractions obtained from the cortex and hippocampus was assessed using a commercial SOD assay kit (cat. no. 19160-1K-F; MilliporeSigma) following the manufacturer’s protocol. Each sample was analyzed in duplicate, and the activity of mitochondrial SOD was adjusted according to mitochondrial protein content and reported as units/mg of mitochondrial protein.

### 2.17. Protein Expression Analysis by Western Blot

Upon completion of the experimental protocol, the expression of COX-2 and IL-6 proteins in cortical and hippocampal tissues was quantified using Western blotting. Brain samples were homogenized in lysis buffer (Thermo Fisher Scientific, Inc.; cat. no. 87792), and total protein concentrations were subsequently determined as previously described [34]. Equal quantities of protein (30 µg per sample) were separated by 10% SDS-polyacrylamide gel electrophoresis and then transferred onto polyvinylidene difluoride (PVDF) membranes (Hybond-P; GE Healthcare Limited, Amersham Place, Little Chalfont, Buckinghamshire, UK). The membranes were incubated overnight at 4 °C with the following primary antibodies: rabbit monoclonal anti-COX-2 (1:1000; ab179800, Abcam, Cambridge, UK), mouse monoclonal anti-IL-6 (1:2000; ab9324, Abcam), and rabbit monoclonal anti-β-actin (1:5000; cat. no. AC026, Abclonal Biotech Co., Ltd., Wuhan, China), which served as the internal loading control. After primary antibody incubation, the membranes were treated with the appropriate horseradish peroxidase-conjugated secondary antibodies, including anti-rabbit (1:2000; cat. no. AS063, Abclonal Biotech Co., Ltd.) or anti-mouse (1:2000; cat. no. 12-349, MilliporeSigma), for 1 h at room temperature. Immunoreactive protein bands were detected using a chemiluminescent substrate (SuperSignal™ West Pico; Pierce, Thermo Fisher Scientific, Inc.). The intensity of the bands was normalized to β-actin to account for variations in protein loading. The relative expression levels of target proteins were quantified using a ChemiDoc™ MP imaging system in combination with Image Lab software (version 6.0.0 build 25; Bio-Rad Laboratories Inc., Hercules, CA, USA).

### 2.18. Statistical Data Analysis

For each experimental time point, differences among the four groups were evaluated independently using a one-way analysis of variance (ANOVA), with Tukey’s multiple-comparison procedure applied when appropriate to identify differences between individual groups. Neurological scores measured in the same animals on Days 1 and 7 were likewise analyzed separately at each time point for comparisons across treatment groups. Changes in neurological scores within individual animals between Day 1 and Day 7 were assessed using the Wilcoxon signed-rank test for paired data. Prior to conducting parametric analyses, the distribution of the data and the assumption of homogeneity of variance were examined. Data are expressed as the mean ± standard error of the mean (SEM). Statistical analyses were conducted using IBM SPSS Statistics version 25 (IBM Corp., Armonk, NY, USA). Statistical significance was defined as *p* < 0.05.

## 3. Results

### 3.1. Naringin Attenuates Neurological Deficits Following Rt.MCAO

Neurological deficit scoring demonstrated that naringin administration effectively mitigated the functional consequences of focal cerebral ischemia (Figure 1). Rats in the sham-operated group exhibited no observable neurological impairment. In contrast, animals subjected to Rt.MCAO and treated with vehicle displayed significantly higher neurological deficit scores both at 1 and 7 days post-ischemia compared with the sham operation group (*p* < 0.05). Naringin treatment significantly improved neurological outcomes, as evidenced by reduced deficit scores at both time points relative to the Rt.MCAO + vehicle (*p* < 0.05). A similar improvement was observed in rats receiving piracetam, which served as a positive control. Furthermore, neurological deficit scores decreased significantly from Day 1 to Day 7 in both the piracetam and naringin groups (*p* < 0.05), indicating significant within-animal improvement in neurological function over the observation period. No significant change between Day 1 and Day 7 was observed in the sham-operated or vehicle-treated groups.

### 3.2. Naringin Reduces Brain Edema Following Rt.MCAO

As illustrated in Figure 2, induction of Rt.MCAO markedly increased brain edema in rats treated with the vehicle compared with the sham operation group (*p* < 0.05). In contrast, administration of naringin significantly attenuated the extent of edema relative to the Rt.MCAO + vehicle group (*p* < 0.05). A comparable reduction was also observed in animals treated with piracetam, indicating a protective effect against ischemia-induced cerebral swelling.

### 3.3. Naringin Reduces Cerebral Infarct Volume After Ischemic Stroke

Brain infarct volume was determined in Rt.MCAO rats to assess the potential protective roles of naringin against ischemic brain injury. Animals received daily intraperitoneal injections of vehicle, piracetam (250 mg/kg BW), or naringin (100 mg/kg BW) for seven consecutive days after ischemic induction. At the end of the treatment period, cerebral infarction was examined using 2,3,5-triphenyltetrazolium chloride (TTC) staining. As illustrated in Figure 3, both piracetam- and naringin-treated groups exhibited a significant reduction in infarct volume compared with the Rt.MCAO plus vehicle group (*p* < 0.05). No infarct area was observed in the sham-operated animals.

### 3.4. Naringin Attenuates Neuronal Loss in the Cerebral Cortex and Hippocampus

Given the beneficial effects of naringin on neurological deficits and cerebral edema, we next investigated whether these functional improvements were associated with enhanced neuronal preservation in ischemia-vulnerable brain regions. Therefore, neuronal density was evaluated in the cerebral cortex and the CA1 and CA3 subregions of the hippocampus. Histological analysis demonstrated that vehicle (DMSO) treatment failed to treat ischemia-induced neuronal loss. In contrast, administration of naringin (100 mg/kg BW) or piracetam (250 mg/kg BW) significantly preserved neuronal density in both the cerebral cortex and hippocampus compared with the Rt.MCAO + vehicle group (*p* < 0.05; Figure 4), suggesting that the neuroprotective effects of naringin are, at least in part, attributable to the attenuation of neuronal degeneration following cerebral ischemia.

### 3.5. Naringin Suppressed Stroke-Induced Microglial Activation in the Cortex and Hippocampus

Microglial activation is a primary hallmark of neuroinflammation. To assess this response, immunohistochemical staining for Iba-1 was conducted on sections from the cortex and hippocampal CA3 region (Figure 5). Ischemic stroke triggered a substantial elevation in both the count of Iba-1-positive microglia and the overall percentage of immunoreactivity (*p* < 0.001), confirming microglial activation. Conversely, administration of piracetam and naringin effectively attenuated this response, significantly reducing the numbers of Iba1-labeled cells in the cortex (*p* < 0.01) and hippocampus (*p* < 0.05). Furthermore, both treatments led to a marked decrease in the immunoreactivity across both brain regions (*p* < 0.001).

### 3.6. Naringin Attenuated Stroke-Induced Astrocyte Activation in the Cortex and Hippocampus

Astrogliosis is a hallmark of post-injury neuroinflammation, and ischemic stroke markedly enhanced GFAP-positive astrocyte activation within the cortex (*p* < 0.001; Figure 6A) and the hippocampal CA3 region (*p* < 0.05; Figure 6B). While administration of piracetam and naringin significantly decreased cortical astrocyte counts (*p* < 0.05), the reduction observed in the hippocampal CA3 did not reach statistical significance. Furthermore, GFAP immunoreactivity was quantified to assess overall expression levels. As shown in Figure 6C,D, the cortex and the hippocampal CA3 exhibited significantly higher immunoreactivity for GFAP in the stroke-vehicle group compared to the sham operation group (*p* < 0.001). However, both piracetam and naringin treatments markedly reduced the expression of GFAP in the cortex (*p* < 0.001) and the hippocampal CA3 (*p* < 0.05) relative to the stroke model.

### 3.7. Naringin Attenuates Myeloperoxidase (MPO) Activity in Ischemic Brain Tissue

Previous findings have demonstrated that induction of Rt.MCAO promotes neuroinflammatory responses that contribute to cerebral injury [30]. To further investigate the involvement of inflammation in ischemic brain damage, MPO activity was evaluated as a marker of neutrophil accumulation and inflammatory activation following ischemic stroke. As presented in Figure 7, MPO activity was significantly elevated in the Rt.MCAO rats receiving vehicle treatment when compared with the control group (*p* < 0.05). However, administration of either naringin or piracetam markedly decreased MPO activity relative to the Rt.MCAO plus vehicle-treated group (*p* < 0.05).

### 3.8. Naringin Modulates Cortical and Hippocampal MDA Levels and Antioxidant Enzyme Activities

To evaluate oxidative damage, MDA, a representative indicator of lipid peroxidation, together with the endogenous antioxidant enzymes CAT, GSH-Px, and SOD, were assessed in the cerebral cortex and hippocampus. Permanent Rt.MCAO markedly disrupted the redox balance, as evidenced by a significant increase in MDA levels accompanied by significant reductions in CAT, GSH-Px, and SOD activities compared with the sham-operated group (*p* < 0.05). Treatment with either piracetam (250 mg/kg BW) or naringin (100 mg/kg BW) significantly attenuated these oxidative alterations. Specifically, both treatments reduced MDA accumulation (Figure 8A) while preserving CAT, GSH-Px, and SOD activities relative to the Rt.MCAO + vehicle group (*p* < 0.05; Figure 8B–D).

### 3.9. Naringin Enhances Mitochondrial Superoxide Dismutase (SOD) Activity in the Cortex and Hippocampus

Mitochondrial SOD activity was markedly reduced in rats subjected to Rt.MCAO compared with the sham-operated animals (*p* < 0.05). However, treatment with either piracetam (250 mg/kg BW) or naringin (100 mg/kg BW) significantly attenuated the decline in mitochondrial SOD activity, resulting in higher enzyme levels than those observed in the vehicle + Rt.MCAO group (*p* < 0.05), as shown in Figure 9.

### 3.10. Naringin Mitigates Neuroinflammation Following Rt.MCAO in Rats

To further clarify the anti-inflammatory mechanism of naringin, the expression levels of cyclooxygenase-2 (COX-2) and interleukin-6 (IL-6) were evaluated, as presented in Figure 10. Western blot analysis was performed to measure protein expression in the cortex and hippocampus. Brain tissues were homogenized in N-PER™ extraction reagent (Thermo Fisher Scientific, Inc.; cat. no. 87792; Waltham, MA, USA) prior to protein assessment. The findings demonstrated that administration of piracetam (250 mg/kg BW) or naringin (100 mg/kg BW) for seven consecutive days significantly decreased COX-2 and IL-6 expression in both the cortex and hippocampus when compared with the Rt.MCAO plus vehicle-treated group (*p* < 0.05).

## 4. Discussion

Cerebral ischemia triggered by middle cerebral artery occlusion (MCAO) activates a cascade of pathological events, including excitotoxicity, oxidative damage, inflammatory activation, and disruption of blood–brain barrier (BBB) integrity, all of which contribute to neuronal loss and subsequent neurological dysfunction [42,43,44]. In the present study, naringin treatment significantly improved neurological function, reduced infarct volume and preserved neuronal density in the cerebral cortex and hippocampus, demonstrating its robust neuroprotective effects against ischemic brain injury. Neurological deficit assessment remains a widely accepted method for evaluating the extent of functional impairment after cerebral ischemia, particularly in areas involved in sensorimotor regulation [45]. Accumulating evidence suggests that citrus flavonoids such as naringin and naringenin exert beneficial effects on vascular endothelial cells through their antioxidant and anti-inflammatory properties [46,47]. These compounds reduce the expression of adhesion molecules, including intercellular adhesion molecule 1 (ICAM-1) and vascular cell adhesion molecule 1 (VCAM-1), thereby limiting leukocyte infiltration and vascular inflammation while enhancing nitric oxide bioavailability to maintain endothelial-mediated vasodilation and cerebral perfusion [28,46,47]. Furthermore, modulation of signaling pathways such as NF-κB and Hippo-YAP may suppress endothelial activation and cellular injury, whereas stabilization of ion channel function appears to reduce apoptotic cell death [45]. Through these coordinated mechanisms, naringin may preserve vascular integrity and contribute to protection against cerebrovascular injury. Therefore, the observed reduction in cerebral edema, together with the preservation of neuronal density, suggests that naringin may attenuate secondary neuronal damage and improve brain tissue preservation following cerebral ischemia. Consistent with the present findings, earlier studies demonstrated that seven days of naringin pre-treatment at 100 mg/kg significantly improved neurobehavioral outcomes following ischemia–reperfusion injury, whereas a lower dose of 50 mg/kg failed to produce similar protective effects [48]. Moreover, evidence from earlier investigations indicates that post-ischemic treatment with piracetam (250–500 mg/kg, i.p.) markedly reduces infarct size [49]. The neuroprotective effects of piracetam have been attributed, in part, to its ability to increase cerebral perfusion and preserve the fluidity of neuronal plasma membranes, thereby supporting normal cellular function under ischemic conditions [50,51,52]. Given its documented neuroprotective effects, particularly its ability to reduce infarct volume and enhance antioxidant defenses after cerebral ischemia, piracetam was selected as the positive control in this study.

Neuroinflammation is a major contributor to secondary brain injury and tissue remodeling following ischemic stroke [20]. Shortly after cerebral ischemia occurs, microglia, the intrinsic immune cells of the central nervous system, become rapidly activated in response to disturbances in brain homeostasis [53]. Once activated, these cells release a variety of pro-inflammatory mediators, including interleukin-1 alpha (IL-1α), tumor necrosis factor alpha (TNF-α), and complement component 1q (C1q), which collectively shape the inflammatory microenvironment within the injured brain [54]. These signaling molecules further induce the transformation of resting astrocytes into the reactive A1 phenotype, a neurotoxic form associated with neuronal degeneration and disruption of neurovascular integrity. During the acute stage of ischemic injury, reactive astrocytes amplify inflammatory responses and aggravate BBB dysfunction, thereby worsening neuronal damage. In contrast, during later stages of recovery, astrocyte proliferation contributes to glial scar formation that serves to confine the lesion and protect adjacent healthy tissue [17,55]. Because of the pivotal role of microglia–astrocyte communication in post-ischemic pathology, targeting this interaction has become an important therapeutic strategy for reducing secondary neuronal damage after stroke. In the present study, both piracetam (250 mg/kg BW) and naringin (100 mg/kg BW) markedly reduced neuroinflammatory responses after ischemic injury, as demonstrated by decreased expression of Iba1 and GFAP in several brain regions. These findings indicate suppression of both microglial activation and reactive astrogliosis, suggesting that the two compounds exert protective effects against glial-mediated secondary injury. Among these agents, naringin, a flavanone glycoside naturally abundant in citrus fruits, has attracted considerable interest because of its broad anti-inflammatory and neuroprotective properties [56]. Previous evidence suggests that naringin modulates key inflammatory signaling pathways, particularly Janus kinase/signal transducer and activator of transcription 3 (JAK/STAT3) and NF-κB, resulting in reduced production of pro-inflammatory cytokines together with increased expression of anti-inflammatory mediators such as IL-10 [57,58]. Through regulation of excessive microglial activation and attenuation of reactive astrocytic responses, naringin may help preserve synaptic organization, reduce progressive neurodegeneration, and improve neurological recovery after ischemic stroke [55,56].

The pathological progression of acute ischemic stroke begins with a sudden interruption of cerebral blood flow, leading to rapid energy failure and subsequent activation of inflammatory cascades within the ischemic penumbra. Among the major mediators involved in this delayed injury process are IL-6 and COX-2, both of which are strongly induced in activated microglia after ischemic insult [59]. Elevated IL-6 levels promote recruitment of inflammatory cells and are closely linked to greater neurological deficits and poorer clinical outcomes [60]. At the same time, COX-2 activity enhances the formation of reactive oxygen species and pro-inflammatory prostanoids, thereby intensifying oxidative stress and lipid peroxidation in vulnerable neural tissue [61]. In agreement with previous studies [48,62], our results demonstrated that naringin treatment significantly suppressed the expression of both IL-6 and COX-2 following ischemic injury. Clinical studies have further shown that patients presenting with elevated IL-6 concentrations during the early phase of stroke are more likely to experience unfavorable long-term functional recovery [63]. Consistent with these observations, the vehicle-treated rats in our study exhibited increased COX-2 and IL-6 expression together with poorer neurological performance, whereas naringin administration reduced COX-2 and IL-6 levels and improved neurological scores. These findings suggest that the neuroprotective actions of naringin are closely associated with suppression of COX-2 and IL-6-mediated inflammatory signaling. By concurrently attenuating inflammatory cytokine activity and oxidative damage, naringin may preserve neuronal structure, reduce ischemia-induced tissue degeneration, and serve as a promising multi-target therapeutic candidate for post-stroke neuroprotection [27].

During the acute phase of permanent ischemic stroke, the abrupt interruption of cerebral blood flow triggers a cascade of pathological events within the affected brain tissue [12]. One of the earliest responses involves activation of microglia and infiltration of neutrophils into both the ischemic core and the surrounding penumbral region [64]. These inflammatory cells release large amounts of MPO, an enzyme that catalyzes the formation of highly reactive oxidants, including hypochlorous acid and reactive nitrogen species (RNS), thereby exacerbating neuronal and vascular injury [65,66]. Concurrently, the deprivation of oxygen and glucose markedly impairs cellular energy metabolism and disrupts mitochondrial function [12,67]. Under these conditions, mitochondria produce excessive superoxide radicals, leading to severe oxidative stress [67]. Under physiological conditions, mitochondrial SOD serves as a critical mitochondrial antioxidant enzyme by converting superoxide radicals into less harmful molecules [68,69]. However, during prolonged ischemic conditions, excessive generation of ROS exceeds the capacity of endogenous antioxidant systems, leading to mitochondrial dysfunction, lipid membrane damage, and ultimately neuronal death through necrotic and apoptotic mechanisms [69]. Recent studies published between 2024 and 2025 have increasingly highlighted the potential of bioactive citrus compounds, particularly naringin, to counteract oxidative stress and inflammatory responses and to preserve cellular and mitochondrial function [28,29,30]. Consistent with these observations, our findings provide further evidence that naringin exerts antioxidant and anti-inflammatory effects in the brain following permanent cerebral ischemia. Naringin treatment was associated with reduced MPO activity and MDA levels, together with decreased expression of inflammatory mediators. In parallel, naringin preserved or enhanced endogenous antioxidant defenses, as reflected by the activities of CAT, GSH-Px, SOD, and mitochondrial SOD in both the cerebral cortex and hippocampus. Taken together, these findings suggest that attenuation of oxidative and inflammatory disturbances may contribute to the neuroprotective effects observed following permanent middle cerebral artery occlusion. Moreover, these results align with earlier research showing that naringin confers neuroprotection against cerebral ischemic injury primarily through the mitigation of oxidative damage and neuroinflammatory responses [21,33,46,70]. Furthermore, naringin attenuates acetaminophen-induced hepatocellular damage by alleviating oxidative stress and maintaining mitochondrial integrity [71]. Collectively, these findings suggest that the combined antioxidant and anti-inflammatory actions of naringin may contribute to protection against ischemia-induced neuronal damage and may help attenuate the progression of irreversible brain injury. Our findings are consistent with previous studies reporting the neuroprotective potential of naringin in experimental models of cerebral ischemia.

While these findings highlight the therapeutic efficacy of post-ischemic naringin intervention, several limitations should be considered. First, naringin was administered intraperitoneally rather than orally to minimize discomfort and avoid irritation of the cervical surgical wound during postoperative recovery. However, this route limits the direct translation of our findings to dietary or oral administration, as naringin undergoes substantial gastrointestinal and hepatic metabolism following oral intake. In addition, plasma and brain concentrations of naringin and its metabolites were not measured. Therefore, the present findings should be considered preliminary evidence of neuroprotective efficacy rather than direct evidence of its effectiveness as a dietary supplement. Future studies using oral administration and pharmacokinetic analyses are needed to determine clinically relevant dosing. Second, only a single dose of naringin (100 mg/kg BW) was evaluated, and the assessment was limited to the acute phase following ischemic injury. Future studies should examine a range of doses and treatment paradigms to identify the optimal therapeutic window and determine whether delayed administration remains effective. Furthermore, naringin markedly attenuated glial activation and was associated with favorable changes in inflammatory and oxidative stress responses, as evidenced by reduced IL-6 and COX-2 expression, decreased MPO activity and MDA levels, and enhanced activities of the antioxidant enzymes CAT, GSH-Px, and SOD. However, the precise molecular mechanisms underlying these effects have not yet been fully characterized. Further investigation is required to identify the upstream signaling pathways and cellular targets involved in naringin-mediated neuroprotection. Third, this study included only male rats, which was intended to reduce potential hormonal variability during this initial proof-of-concept investigation. However, sex is an important factor influencing ischemic brain injury, neuroinflammatory responses, and oxidative stress. Therefore, the present findings may not be directly applicable to female subjects. Further studies using both male and female animals, including aged females and ovariectomized models, are needed to determine whether the neuroprotective effects of naringin are consistent across sexes and to better assess its potential for clinical translation. Finally, the current study focused primarily on acute outcomes. Long-term behavioral and functional assessments will be important to establish whether the observed neuroprotective effects translate into sustained neurological recovery and protection against chronic post-stroke neurodegeneration.

The present study demonstrates that naringin may counteract several pathological consequences of permanent cerebral ischemia. Treatment was associated with better neurological outcomes, smaller infarct areas, reduced cerebral edema, and greater preservation of neuronal populations in the cortex and hippocampus. At the molecular and cellular levels, these benefits coincided with diminished glial reactivity and inflammatory signaling, as well as reduced lipid peroxidation and maintenance of antioxidant enzyme activity. The simultaneous improvement in antioxidant capacity and suppression of neuroinflammatory responses suggests that naringin may mitigate secondary ischemic damage through complementary protective actions. Taken together, these findings strengthen the evidence supporting naringin as a potential neuroprotective compound and provide a basis for further investigation of its therapeutic relevance in cerebral ischemia.

## 5. Conclusions

In conclusion, naringin treatment was associated with improved neurological function, reduced infarction and brain edema, and preservation of neuronal integrity following permanent cerebral ischemia in rats. These effects coincided with enhanced antioxidant defenses and reduced oxidative and neuroinflammatory responses. However, further preclinical studies are needed to confirm the underlying mechanisms, optimal treatment conditions, and long-term effects. Subsequent clinical studies will also be necessary to determine whether these findings can be translated into a potential therapeutic application.

## Figures and Tables

**Figure 1:**
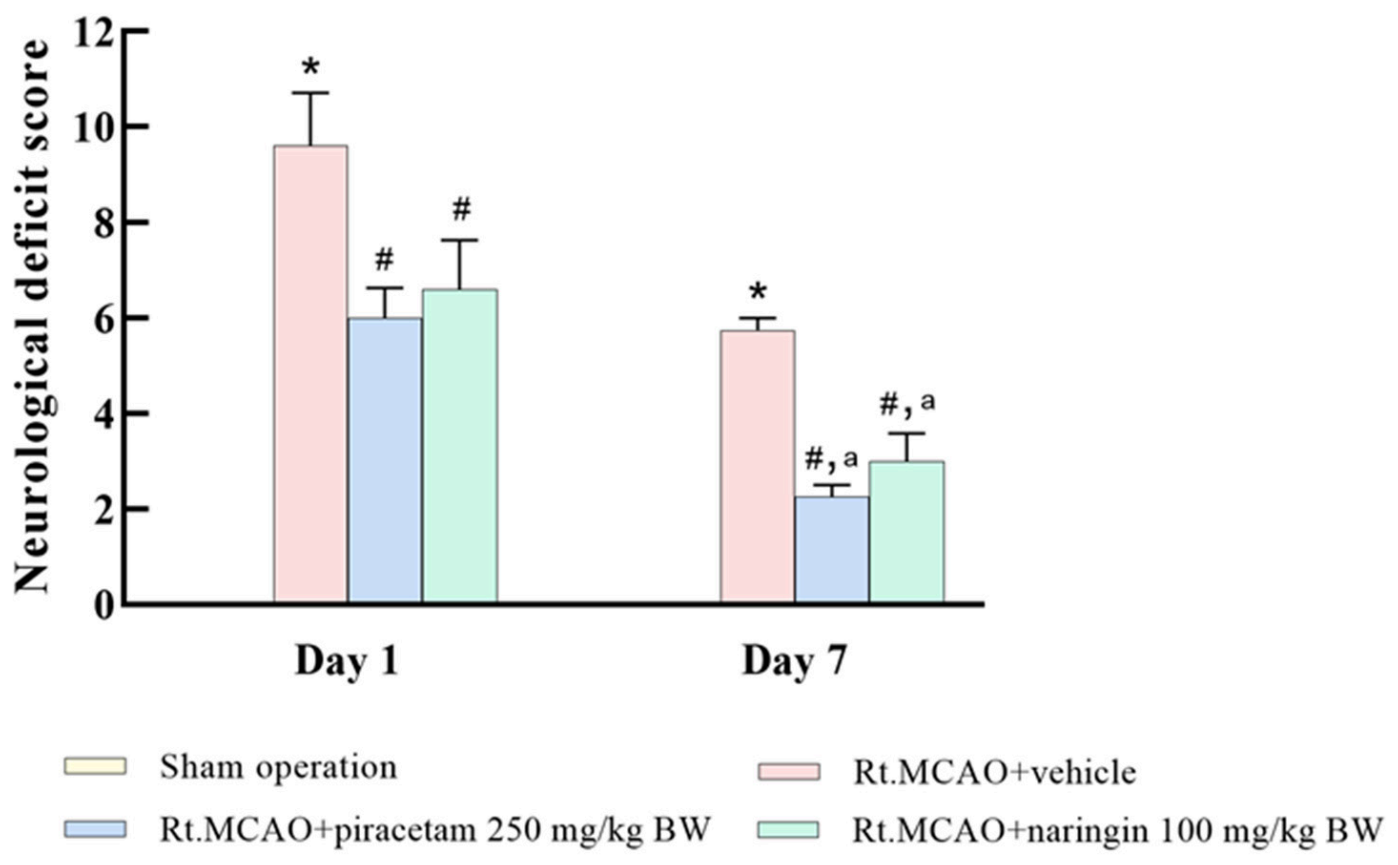
Effects of naringin on neurological deficit score. Data are expressed as the mean ± SEM (n = 5). * *p* < 0.05 compared with the sham-operated group; ^#^ *p* < 0.05 compared with the Rt.MCAO + vehicle group; ^a^ *p* < 0.05 compared between day 1 and day 7. Rt.MCAO: right middle cerebral artery occlusion; BW: body weight.

**Figure 2:**
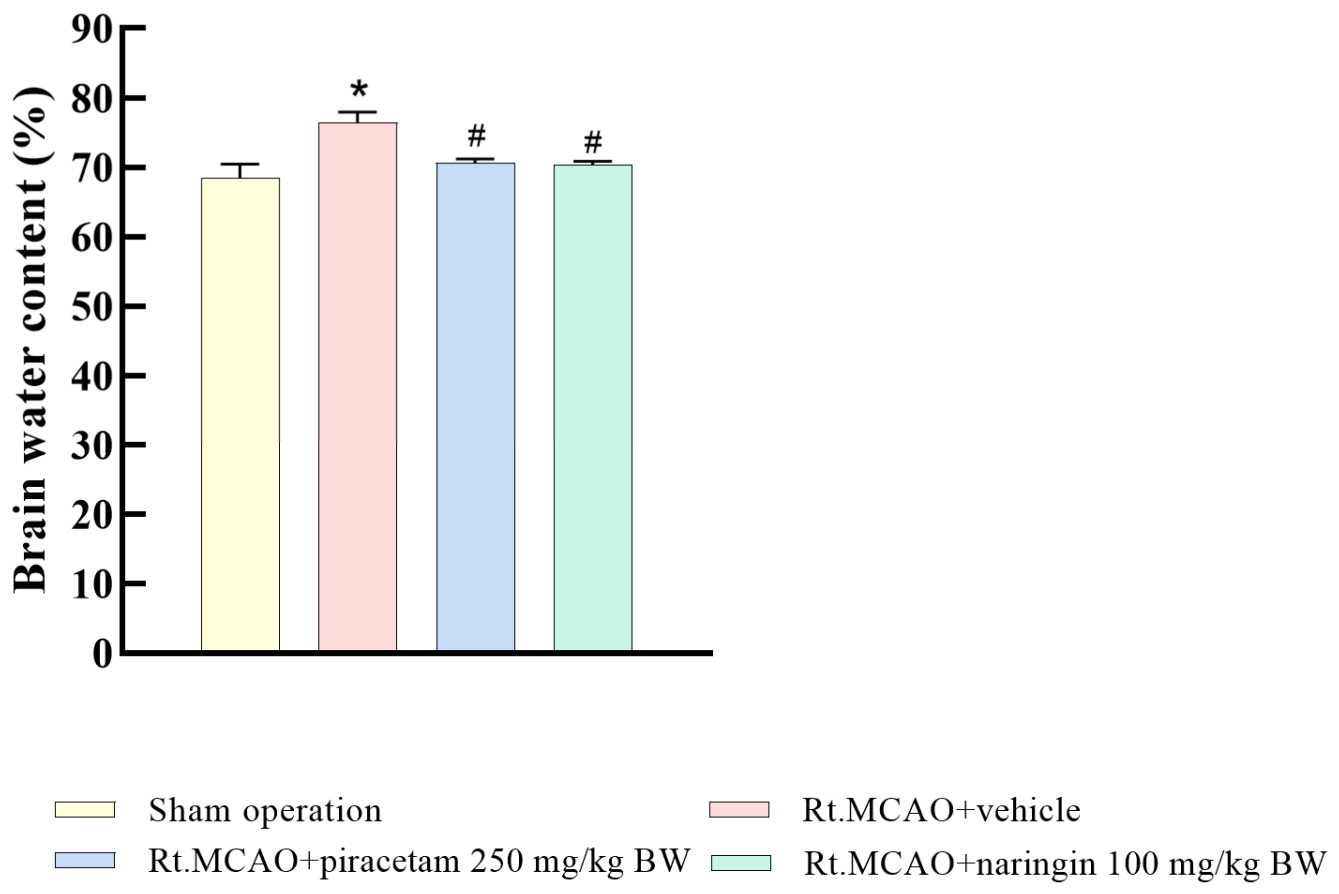
Effects of naringin on brain water content. Data are expressed as the mean ± SEM (n = 5). * *p* < 0.05 compared with the sham-operated group; ^#^ *p* < 0.05 compared with the Rt.MCAO + vehicle group. Rt.MCAO: right middle cerebral artery occlusion; BW: body weight.

**Figure 3:**
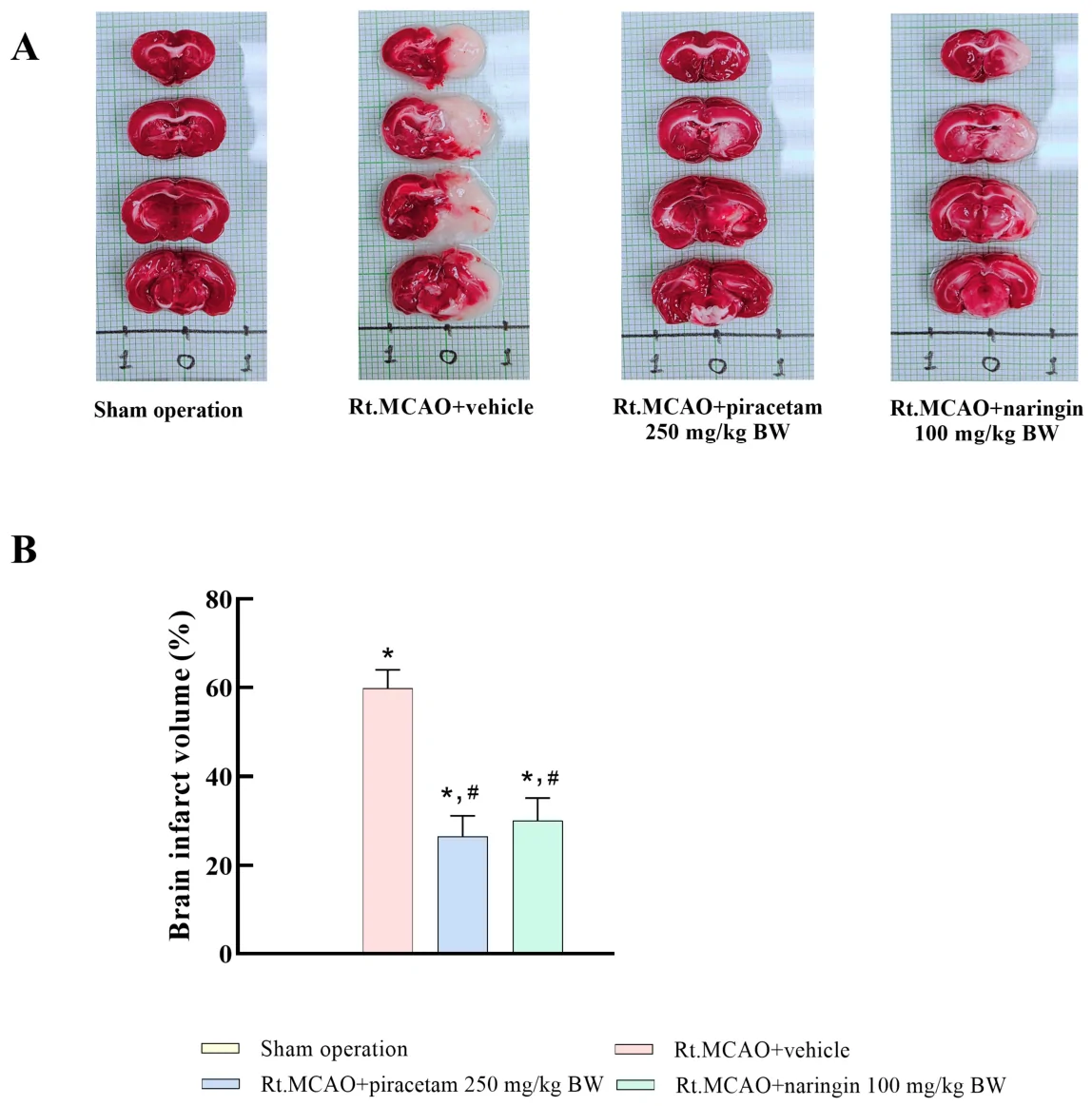
Effect of naringin on cerebral infarct volume. (**A**) Representative TTC-stained coronal brain sections from each experimental group. Red-stained regions represent viable brain tissue, whereas pale or unstained regions indicate infarcted tissue. (**B**) Quantitative analysis of infarct volume in rats subjected to cerebral ischemia. Results are presented as mean ± S.E.M. (n = 5). * *p* < 0.05 versus the sham-operated group; ^#^ *p* < 0.05 versus the Rt.MCAO plus vehicle-treated group. Rt.MCAO: right middle cerebral artery occlusion; BW: body weight.

**Figure 4:**
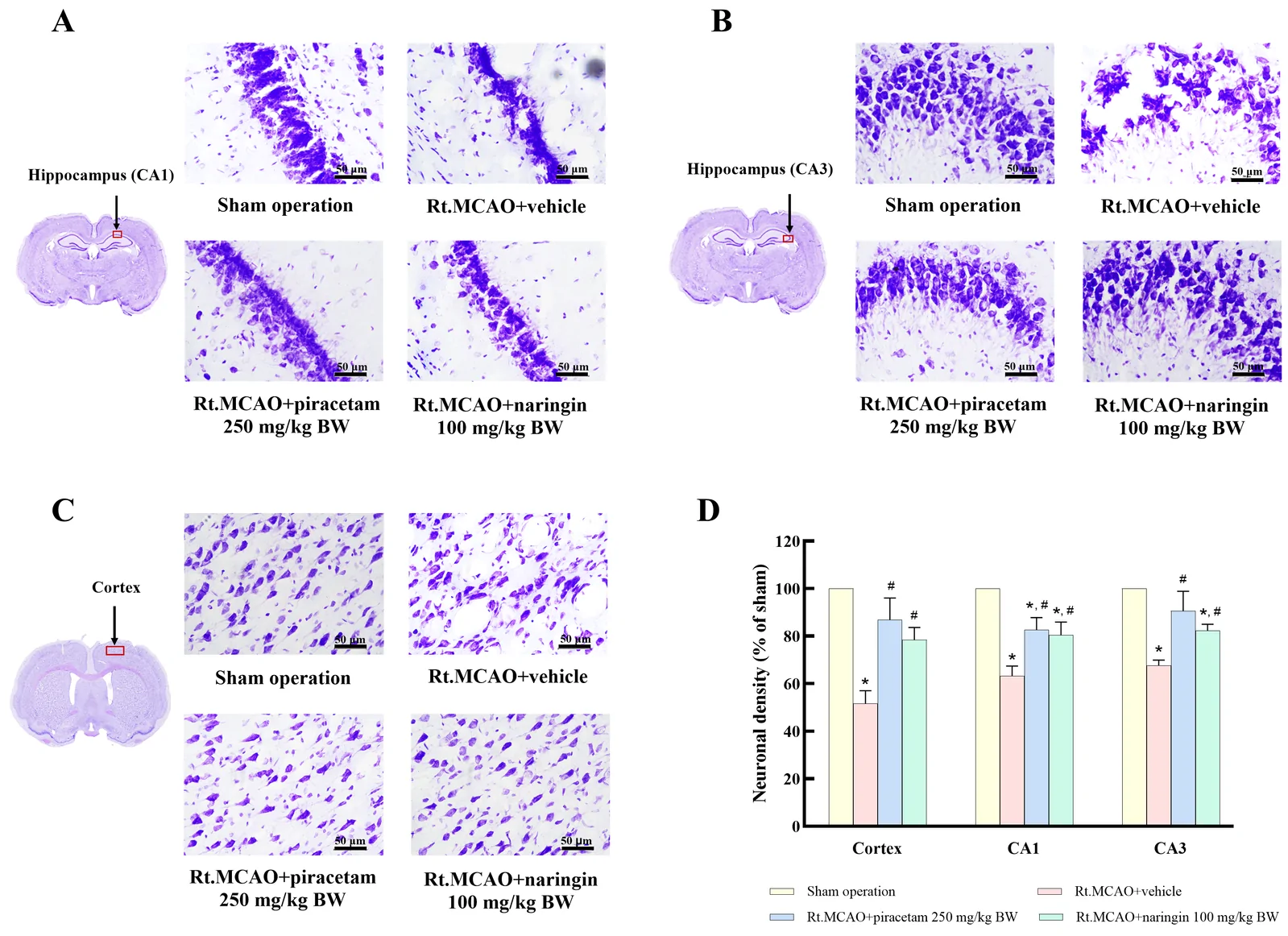
Effects of naringin on neuronal density in the cerebral cortex and hippocampus following permanent Rt.MCAO. (**A**–**C**) Representative photomicrographs of Nissl-stained brain sections showing the CA1 and CA3 subregions of the hippocampus and cerebral cortex (40× magnification). (**D**) Quantitative analysis of neuronal density in the cerebral cortex and the CA1 and CA3 hippocampal subregions. Data are presented as the mean ± SEM (n = 5). * *p* < 0.05 versus the sham-operated group; ^#^ *p* < 0.05 versus the Rt.MCAO plus vehicle-treated group. Rt.MCAO, right middle cerebral artery occlusion; BW, body weight.

**Figure 5:**
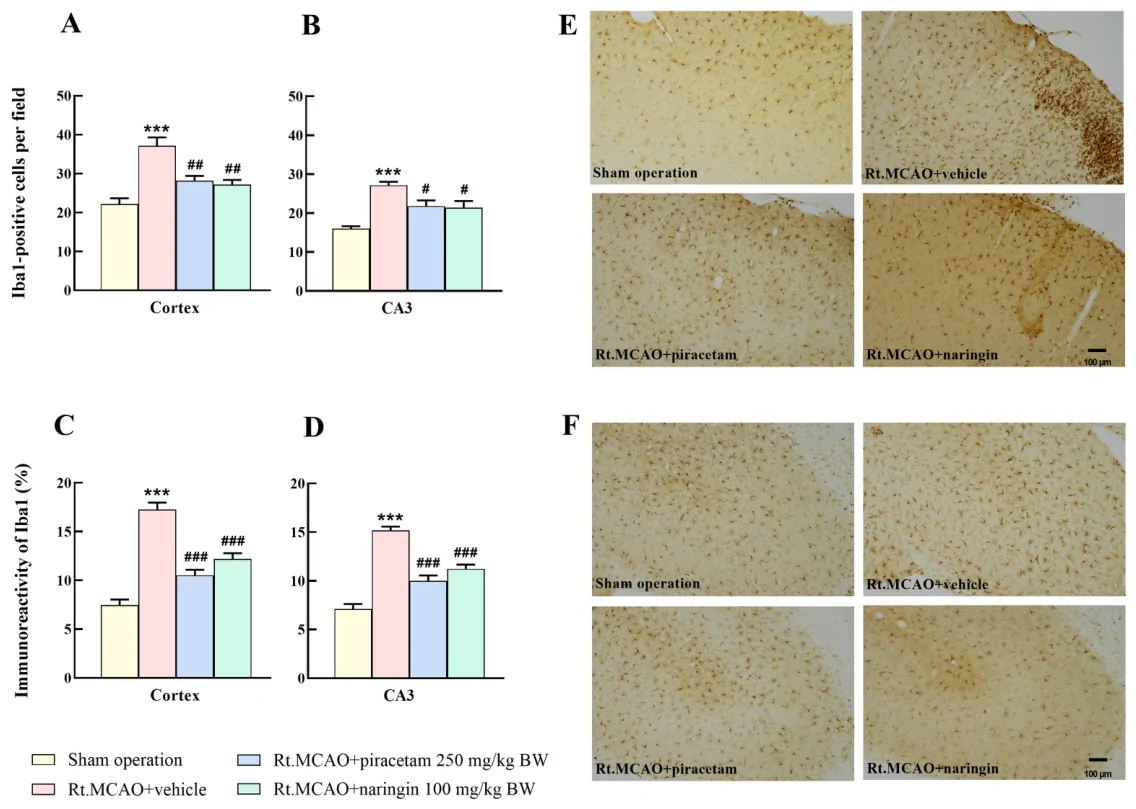
Effect of naringin on microglial activation detected by Iba-1 immunohistochemical staining in the cortex and hippocampus. The number of Iba1-positive cells (**A**,**B**). The immunoreactivity of Iba-1 staining (**C**,**D**). Representative images of Iba-1 labeling within the cortex (**E**) and the hippocampal CA3 (**F**). Data are expressed as the mean ± SEM (n = 5). *** *p* < 0.001 compared with the sham-operated group; ^###^ *p* < 0.001, ^##^ *p* < 0.01, ^#^ *p* < 0.05 compared with the Rt.MCAO + vehicle group; Rt.MCAO: right middle cerebral artery occlusion; BW: body weight. The scale bar represents 100 µm.

**Figure 6:**
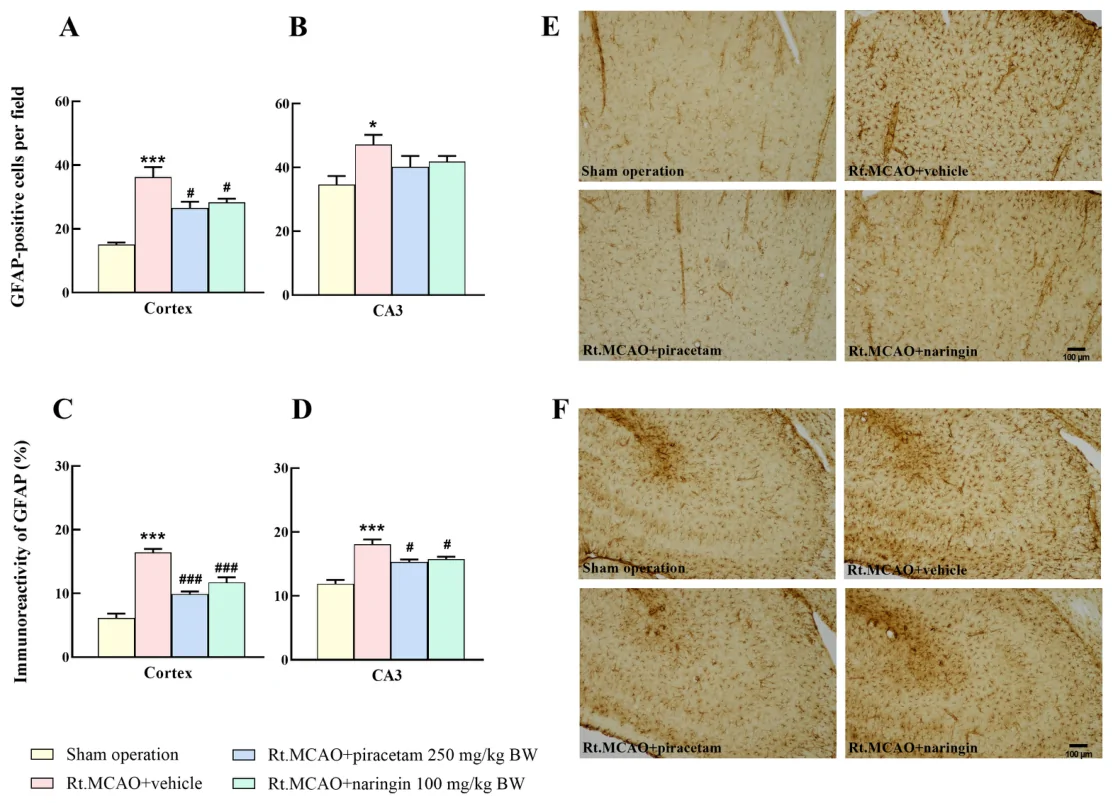
Effect of naringin on astrocyte activation detected by GFAP immunohistochemistry in the cortical and hippocampal tissues. The quantification of GFAP-positive cells (**A**,**B**). The immunoreactivity of GFAP staining (**C**,**D**). Representative images of GFAP labeling within the cortex (**E**) and hippocampal CA3 (**F**). Values are reported as the mean ± SEM (n = 5). * *p* < 0.05, *** *p* < 0.001 compared with the sham-operated group; ^###^ *p* < 0.001, ^#^ *p* < 0.05 compared with the Rt.MCAO + vehicle group; Rt.MCAO: right middle cerebral artery occlusion; BW: body weight. The scale bar represents 100 µm.

**Figure 7:**
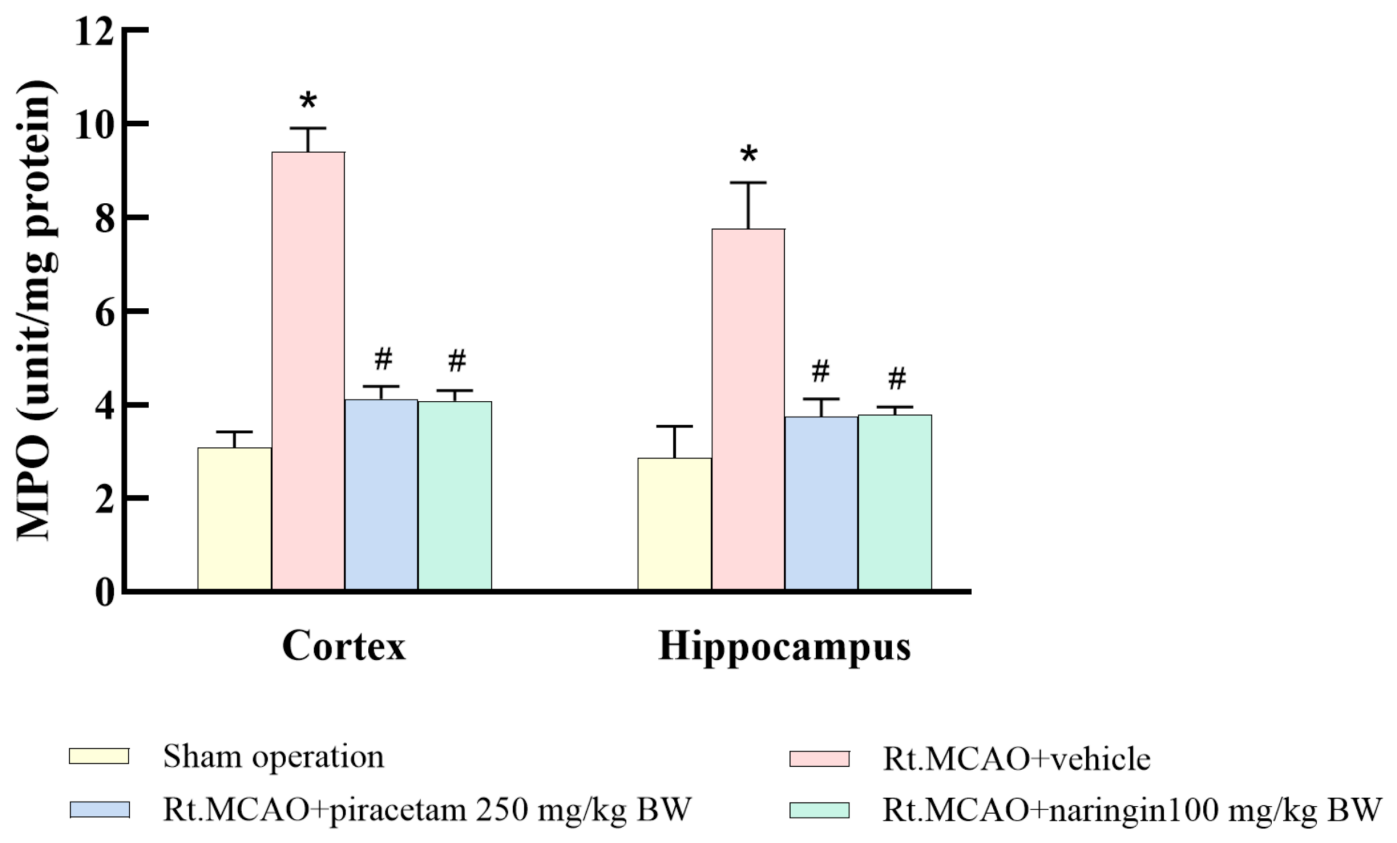
The effects of naringin treatment on MPO levels were evaluated in cortical and hippocampal tissues obtained from Rt.MCAO-induced rats. Results are presented as mean ± S.E.M. for groups of five animals (n = 5). Statistical significance (*p* < 0.05) is indicated by an asterisk (*) compared with the sham-operated group and by a pound sign (^#^) compared with the vehicle-treated Rt.MCAO group. BW: body weight.

**Figure 8:**
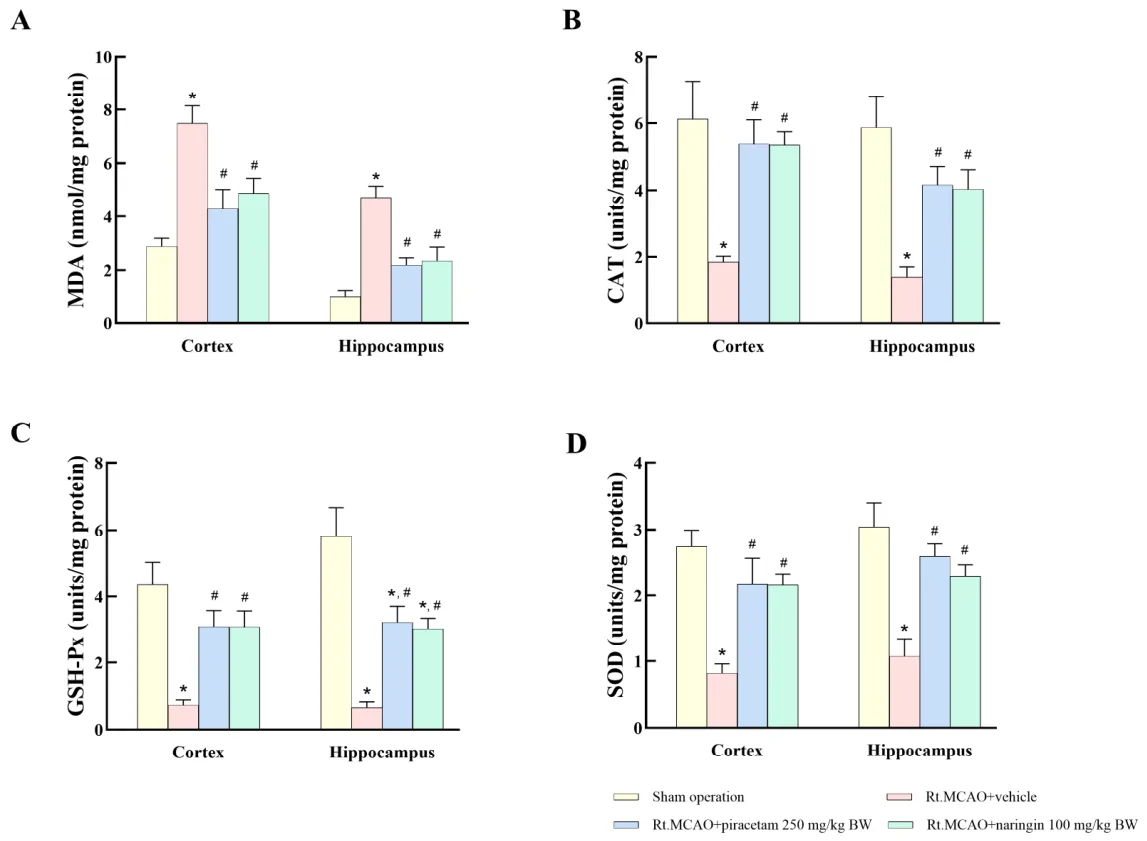
Effects of naringin on oxidative stress markers and antioxidant enzyme activities in the cerebral cortex and hippocampus. (**A**) Malondialdehyde (MDA) levels. (**B**) Catalase (CAT) activity. (**C**) Glutathione peroxidase (GSH-Px) activity. (**D**) Superoxide dismutase (SOD) activity. Data are presented as the mean ± SEM (n = 5). * *p* < 0.05 versus the sham-operated group; ^#^ *p* < 0.05 versus the Rt.MCAO + vehicle group.

**Figure 9:**
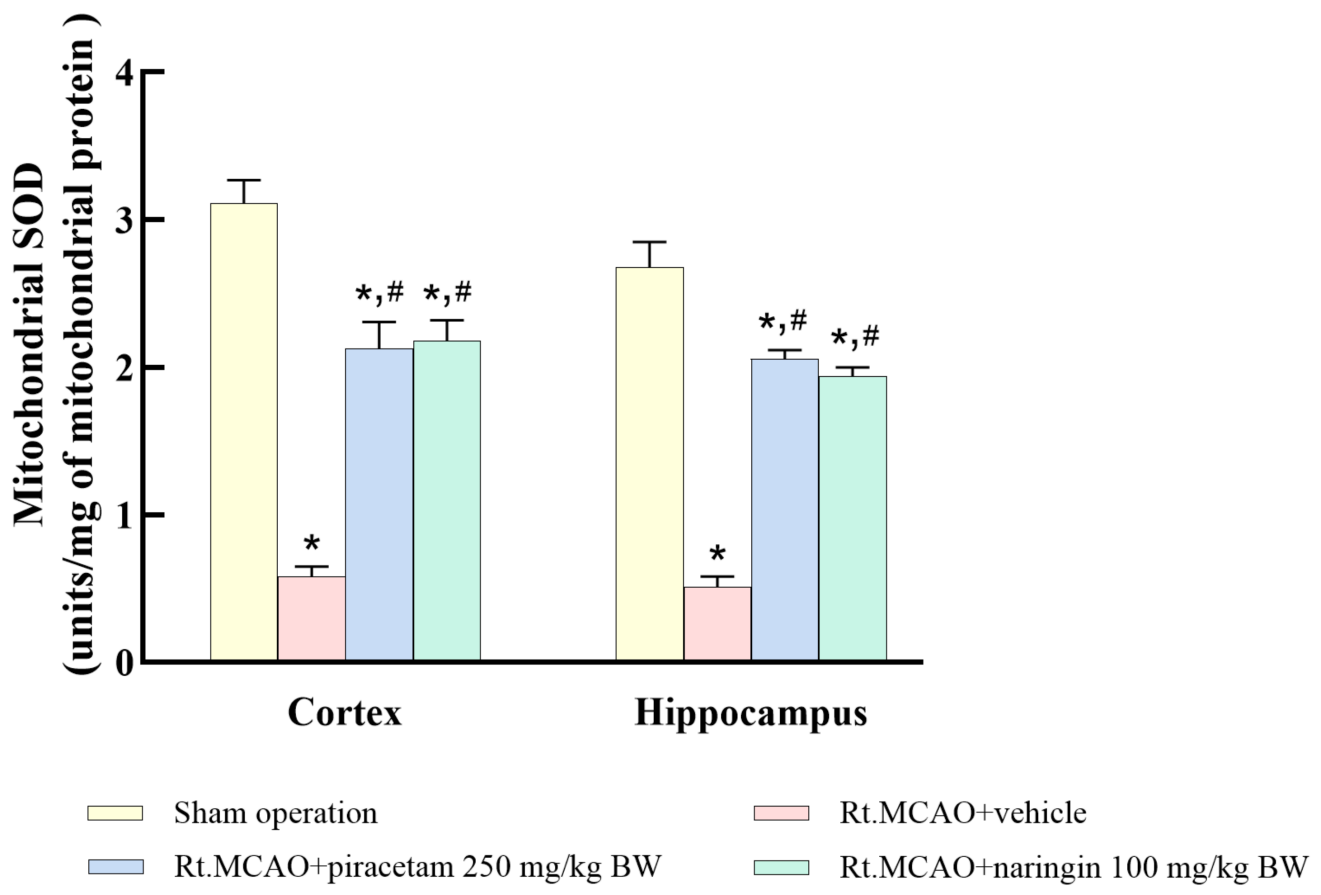
The activity of mitochondrial superoxide dismutase (SOD) was evaluated in the cortical and hippocampal regions of rats after Rt.MCAO induction and subsequent administration of vehicle, piracetam, or naringin. Values are expressed as mean ± S.E.M. (n = 5). * *p* < 0.05 compared with the sham-operated group, whereas ^#^ *p* < 0.05 compared with the Rt.MCAO plus vehicle-treated group. BW: body weight.

**Figure 10:**
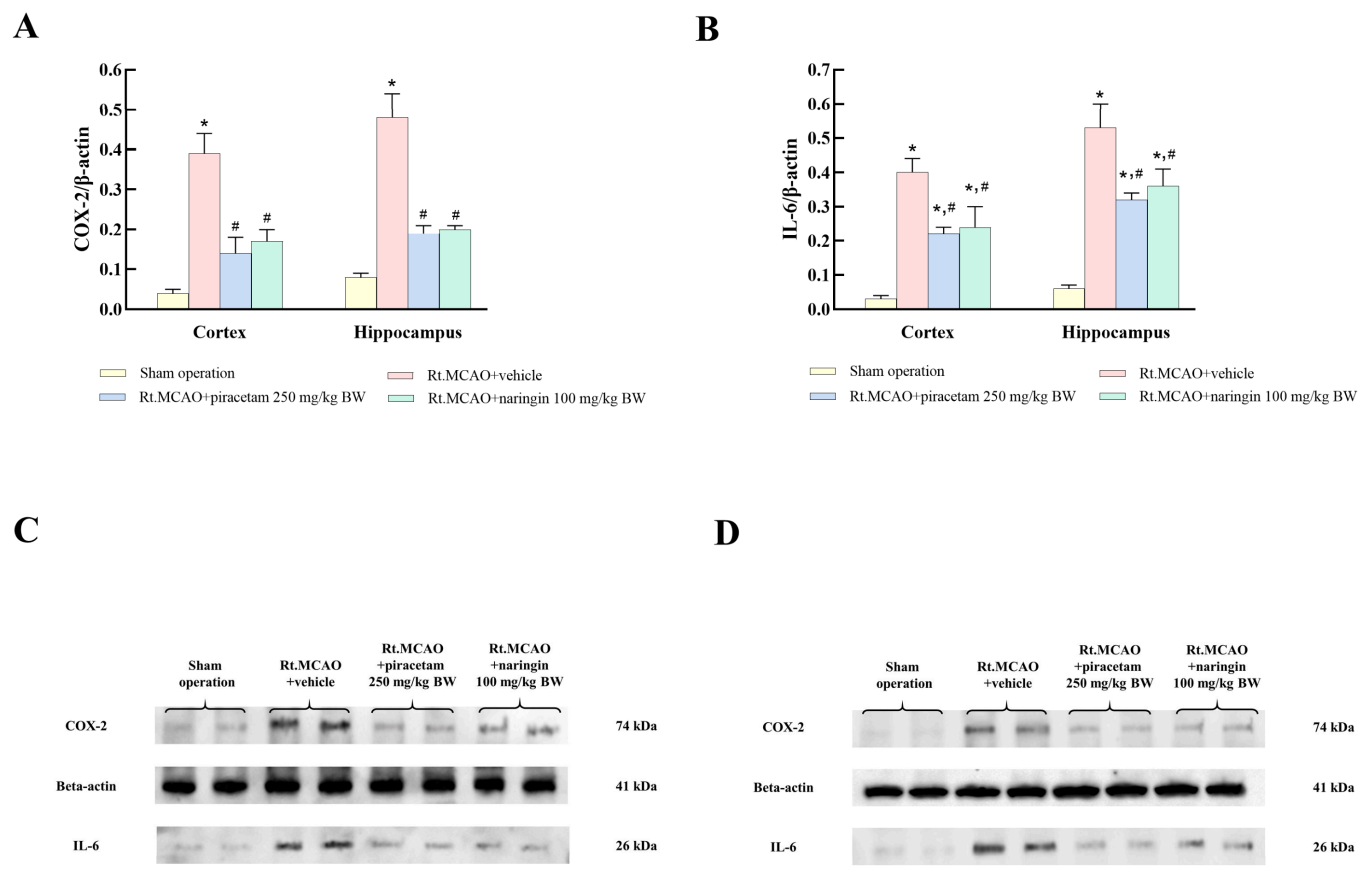
Neuroprotective effects of naringin against Rt.MCAO-induced cerebral injury through modulation of inflammatory signaling pathways. (**A**,**B**) Quantitative densitometric analyses of (**A**) COX-2 and (**B**) IL-6 protein expression levels. (**C**,**D**) Representative immunoblot images showing COX-2 (74 kDa) and IL-6 (26 kDa) expression in the cortex and hippocampus. β-Actin (41 kDa) served as the internal loading control. Data are presented as mean ± SEM (n = 5 per group). * *p* < 0.05 compared with the sham-operated group; ^#^ *p* < 0.05 compared with the Rt.MCAO + vehicle-treated group.

**Table 1:** Scoring criteria used for neurological assessment with the 20-point modified neurologic severity score.

Test Category	How to Test	Point
Movement and energy (spontaneous motility)	• The rat walks normally and explores its cage. • The rat moves around, but shows less interest or curiosity than usual. • The rat moves its legs but stays in one place without walking forward. • The rat stays totally still; it only moves if you touch or nudge it. • The rat does not respond to touch, but its muscles are still normal (not limp). • The rat is completely weak, its muscles are totally limp, or it is near death.	0 1 2 3 4 5
2. Walking patterns (gait disturbances)	• The rat walks completely straight and normal. • The rat drifts or leans slightly toward its healthy side while walking. • The rat switches between walking straight and walking in circles. • The rat switches between circling and leaning toward its weak (paretic) side. • The rat circles constantly and has severe trouble walking. • The rat circles non-stop toward its weak (paretic) side.	0 1 2 3 4 5
3. Tail suspension test	Hold the rat gently by the base of its tail and lift it off the ground: 3.1 Front Paw Bending: Check if the paw on the stroke side curls inward (0 = normal, 1 = mild bend, 2 = tight curl). 3.2 Body Twisting: Check if the rat’s torso twists and rotates in the air (0 = straight body, 1 = mild twist, 2 = severe twist).	0–2 0–2
4. Side balance (lateral push)	Place the rat on a flat surface and give its shoulder a gentle push from the side: • Measures how well the rat stands its ground and keeps its balance without slipping or falling over (0 = strong balance, 1 = weak balance, 2 = falls over).	0–2
5. Paw placing (reflex testing)	Hold the rat and let the back of its paws touch the edge of a table to see if it lifts and places its paw down on top of the surface: 5.1 Healthy Side Paw: Normal, weak, or zero placing reflex. 5.2 Stroke Side Paw: Normal, weak, or zero placing reflex.	0–2 0–2
	**Total Score Summary**	20

## Data Availability

The original contributions presented in this study are included in the article. Further inquiries can be directed to the corresponding author.

## Abbreviations

The following abbreviations are used in this manuscript:

ROS	Reactive oxygen species
SOD	Superoxide dismutase
CAT	Catalase
GSH-Px	Glutathione peroxidase
NF-κB	Nuclear factor kappa B
COX-2	Cyclooxygenase-2
IL-6	Interleukin-6
Iba1	Ionized calcium-binding adapter molecule-1
BBB	Blood–brain barrier
DMSO	Dimethyl sulfoxide
Rt.MCAO	Right middle cerebral artery occlusion
i.p.	Intraperitoneal
mg	Milligram
kg	Kilogram
BW	Body weight
mNSS	Modified neurologic severity score
MCA	Middle cerebral artery
TTC	2,3,5-triphenyltetrazolium chloride
MDA	Malondialdehyde
MPO	Myeloperoxidase
PBS	Phosphate-buffered saline
PFA	Paraformaldehyde
TNB	5-thio-2-nitrobenzoic acid
TBA	Thiobarbituric acid
H_2_O_2_	Hydrogen peroxide
NADPH	Nicotinamide adenine dinucleotide phosphate
PVDF	Polyvinylidene difluoride
ANOVA	Analysis of Variance
SPSS	Statistical Package for the Social Sciences
MCAO	Middle cerebral artery occlusion
ICAM-1	Intercellular adhesion molecule 1
VCAM-1	Vascular cell adhesion molecule 1
IL-1α	Interleukin-1 alpha
TNF-α	Tumor necrosis factor alpha
C1q	Complement component 1q
JAK	Janus kinase
STAT3	Signal transducer and activator of transcription 3
IL-10	Interleukin-10
RNS	Reactive nitrogen species
